# A new approach to categorization of radiologic inflammation in chronic rhinosinusitis

**DOI:** 10.1371/journal.pone.0235432

**Published:** 2020-06-29

**Authors:** Jordan R. Kuiper, Annemarie G. Hirsch, Karen Bandeen-Roche, Agnes S. Sundaresan, Bruce K. Tan, Robert C. Kern, Robert P. Schleimer, Brian S. Schwartz

**Affiliations:** 1 Department of Environmental Health and Engineering, Johns Hopkins University Bloomberg School of Public Health, Baltimore, Maryland, United States of America; 2 Department of Population Health Sciences, Geisinger, Danville, Pennsylvania, United States of America; 3 Department of Biostatistics, Johns Hopkins University Bloomberg School of Public Health, Baltimore, Maryland, United States of America; 4 Department of Otolaryngology Head and Neck Surgery, Northwestern University Feinberg School of Medicine, Chicago, Illinois, United States of America; 5 Division of Allergy and Immunology, Department of Medicine, Northwestern University Feinberg School of Medicine, Chicago, Illinois, United States of America; University of Queensland Centre for Clinical Research, AUSTRALIA

## Abstract

Chronic rhinosinusitis (CRS) is a prevalent condition. Clinical diagnosis requires subjective evidence (i.e. symptoms) and objective evidence of inflammation (e.g. sinus computed tomography [CT]). Few studies have assessed differences in common CT scoring approaches for CRS, the Lund-Mackay (LM) system and its modified version (mLM); none in a general population sample. The aims of this study were to answer the following: (1) Is mLM superior to LM? (2) Should nasal cavity opacification be included in scoring? (3) How should location-specific scores be utilized? (4) If location-specific scores are summed, what should be the cutoff? (5) Are associations of opacification with symptoms observed when using different measurement approaches? We scored sinus CTs using LM and mLM from 526 subjects selected from a larger CRS study. Exploratory factor analysis (EFA) assessed similarity of mLM and LM. Latent class analysis (LCA) identified subgroups of sinus opacification patterns. Factors associated with group membership and relations with nasal and sinus symptoms (NSS) guided clinical relevance. EFA suggested no differences between LM and mLM, or after addition of nasal cavity opacification. LCA identified three opacification groups: no/mild, localized, and diffuse. Males were 2.7x more likely to have diffuse opacification than females, as were those with asthma or hay fever. A LM cutoff of 3 had similar performance to the currently used 4. Diffuse opacification was associated with nasal blockage and smell loss. Differing patterns of opacification may be clinically relevant, improving measurement of objective evidence in studies of CRS and sinus diseases.

## Introduction

Chronic rhinosinusitis (CRS) is a prevalent condition of the paranasal sinuses [1]. Clinical diagnosis of CRS, suggested by American [2], European [1], and international [3] consensus groups, requires two components: 1) objective evidence of sinus inflammation by imaging (e.g. computed tomography [CT] scan) or nasal endoscopy, and 2) subjective evidence of self-reported nasal and sinus symptoms (NSS). Considering the difficulty and cost associated with obtaining objective evidence of inflammation, EPOS has also proposed a symptom-based CRS definition (CRS_S_) to be used in epidemiologic research [1].

The Lund-Mackay (LM) [4] scoring approach is recommended for CRS [5] and measures opacification in three categories (none, partial, total) in five sinuses (maxillary, anterior and posterior ethmoids, frontal, and sphenoid) as well as the osteomeatal complex (OMC, none or total), with individual scores summed to a total of 0–24. A previous study of tertiary care patients with indications requiring sinus CT scans suggested a total score of LM < 4 be used as the cutoff for determining “normal” sinus opacification with low probability for sinus disease [6]. The modified LM (mLM) [7, 8] is a variant of LM with finer gradation in sinus opacification (five categories) allowing for a total score of 0–44. Neither approach includes nasal cavity opacification as part of the total score, despite the fact that nasal polyposis, usually detected through endoscopic examination of the nasal cavity, causes nasal cavity opacification, and characterizes one phenotype of CRS (CRS with nasal polyps [CRSwNP]) [1]. While nasal polyps generally develop in the anterior and posterior ethmoids, not all individuals with opacification in the ethmoids go on to develop nasal polyps [9, 10]. As such, inclusion of nasal cavity opacification in scoring approaches may provide important information on disease severity and prognostication.

It has been well-documented that objective and subjective evidence of CRS correlate poorly [5, 11–13]; however, there are many potential reasons for the observed inconsistencies. NSS are common to several conditions e.g., acute rhinosinusitis (ARS), allergic rhinitis, migraine headache; therefore, individuals with these morbidities often meet criteria for CRS_S_ while having no objective evidence of sinus inflammation. Further, symptom scoring approaches like CRS_S_ consider NSS to be largely interchangeable; however, a recent study of NSS has shown the four symptom groups used in CRS_S_ (nasal blockage/congestion/obstruction; anterior/posterior nasal discharge; smell loss; and facial pain/pressure), identify separate constructs, not one, which would be expected if they all represent the same underlying process or pathogenesis [14]. Additionally, few studies have assessed sinus opacification in a general population [6, 10, 15] and only one among a sample without indications requiring a CT scan of the head [10]. As such, there have not been rigorous approaches to measurement across the broad spectrum of disease. Considering causation, inflammation causes manifestation of symptoms, not vice versa. Given the above, it is not surprising that NSS cannot be used to identify those likely to have sinus opacification. Given the widespread use of LM for determining extent of sinus disease and as part of criteria for classifying individuals with CRS in research settings, this study had five primary objectives: 1) evaluate two common scoring approaches, mLM and LM, to determine whether mLM had better measurement properties compared to LM; 2) given that nasal polyps eventually extend to the nasal cavity, determine whether addition of nasal cavity opacification impacted scoring approaches; 3) assess whether sinus scores should be summed into one index, as current scoring proposes, or whether other approaches to measurement provide additional information; 4) if sinus locations are to be summed, determine if the current cutoff of LM ≥ 4 is suggested based on a general population sample; and 5) based on findings 1–4, determine whether new approaches to measurement of radiologic inflammation are associated with NSS.

## Materials and methods

### Study overview and participant selection

We completed sinus CT scans on 646 subjects selected from a larger longitudinal study of CRS epidemiology. Details of the longitudinal study have been published elsewhere [16, 17]. Briefly, in 2014, primary care patients at least 18 years of age were selected (n = 23,700) from the electronic health record (EHR) of Geisinger, a health system in Pennsylvania and New Jersey whose primary care population is representative of the general population [18], to participate in the longitudinal study (n = 7847 responders at baseline).

We used a stratified-random sampling approach to over-sample individuals with NSS as well as racial/ethnic minorities. As such, while study participants included the wide spectrum of NSS and sinus disease, individuals with NSS and diagnosis and procedure codes relevant to NSS and sinus disease were more likely to be invited to participate in the sinus CT scan study. Questionnaires were sent to subjects prior to their scheduled CT visit. Subjects who were pregnant were excluded and subjects reporting a cold or upper respiratory infection in the past 30 days were asked to postpone their CT visit. Of the 3269 subjects invited to participate, 646 completed a sinus CT, but this 19.8% participation rate did not appear to induce selection bias as the sample was comparable to the larger study cohort [10]. The sample size was determined primarily by budgetary limits as key parameters needed for sample size calculations were not available before this study. Additionally, we used a data-driven approach to our analysis.

This study was approved by Geisinger’s Radiation Safety Committee and Institutional Review Board (IRB), which has an IRB Authorization Agreement with the Johns Hopkins Bloomberg School of Public Health. Health Insurance Portability and Accountability Act authorization was approved and written informed consent was received from all participants [10].

### CT imaging, staging, and scoring

We completed low-dose radiation, non-contrast sinus CT scans (coronal 3mm slices) for all study subjects, with an estimated effective dose less than 0.15 mSv. We de-identified CT images and they were then independently assessed by two otorhinolaryngologists who were blinded to CRS symptoms. CT images were scored using the mLM scoring approach [8]. All locations were scored separately for the left and right sides with the OMC scored from 0–2 (0 = no occlusion, 1 = partial occlusion, 2 = complete occlusion) and sinuses scored from 0–4 (0 = 0% opacification, 1 = 1–33%, 2 = 34–66%, 3 = 67–99%, or 4 = 100%). Nasal cavity opacification was also assessed with a score from 0–4 (0 = none, 1 = above middle turbinate, 2 = above inferior turbinate, 3 = at or below inferior turbinate, 4 = total opacification) per side. We used average scores when otorhinolaryngologists differed by one point and required adjudication by discussion and agreement for scores differing by greater than one point. We also converted mLM scores to traditional LM scores [4, 5] (sinuses: 0 = no opacification, 1 = partial opacification, 2 = complete opacification; OMC: 0 = no occlusion and 2 = occlusion). The sum of scores for all six locations for both sides became the total LM score [19]. Lastly, CT readers recorded evidence of prior sinus surgery based on CT imaging.

### CRS symptoms and CRS_S_ index

Subjects reported the frequency of CRS_S_ symptoms (four symptom groups) in the past three months using a five-point Likert scale (0 = “never”, 1 = “once in a while”, 2 = “some of the time”, 3 = “most of the time”, and 4 = “all of the time”). We assessed symptoms individually and combined as a CRS_S_ index to indicate overall NSS burden. The index followed the same logic of CRS_S_ and collapsed the six symptoms into four symptom groups: nasal blockage; nasal discharge (average of self-reported nasal discharge and post-nasal drip); smell loss; facial symptoms (average of self-reported facial pain and pressure). We summed these four symptom groups to create a score ranging from 0–16.

### Exploratory factor analysis

To evaluate whether the current recommendation to sum all six locations was justified, we first applied exploratory factor analysis (EFA) to evaluate whether all locations tended to opacify together in smaller groupings [20]. We used maximum likelihood estimation for all models and oblique oblimin rotation to allow correlation among extracted factors. The number of extracted factors was chosen based on eigenvalues of factors [20]; sample-size adjusted Bayesian information criterion (SSABIC) [21]; and inspection of scree-plots [20] (S1 Fig and S1 Table). As a sensitivity analysis, we additionally included nasal cavity opacification as a binary variable (0 = score less than one; 1 = score of at least one). To assess the influence of few, large mLM/LM scores, we estimated models using categorized versions of the original scoring approaches and polychoric (or tetrachoric) correlations. We collapsed mLM scored locations into three categories: 0, 1, and > 1 and dichotomized LM scored sinus locations (0 and ≥ 1).

### Latent class analysis

We conducted latent class analysis (LCA) [20, 22] to identify potential subgroups of individuals based on sinus opacification patterns, as they may have clinical significance and inform pathogenesis. Whereas EFA determines how sinus opacification variables “cluster”, LCA determines how people cluster by their patterns of opacification. For model identifiability, binary indicators of LM sinus scores were used in analyses (0 and ≥ 1). Increasing numbers of classes were fit and compared to guide the final LCA solution. We determined appropriateness of LCA model fit primarily by Lo-Mendell-Rubin likelihood ratio test (LRT) [23, 24] and bootstrapped LRT [25]; however, we also compared frequencies of patterns of opacification co-occurrence expected from the estimated LCA models and observed in the data, with reduced discrepancies (as determined by standardized residuals) indicating improved model fit. Posterior probabilities of opacification (π) were used to interpret the classes [20].

### Risk factors for radiologic inflammation latent classes and symptom burden

We obtained demographic information, smoking status (never, former, current), and comorbidities used in the creation of the Charlson comorbidity index [26] from the EHR. The anxiety sensitivity index (ASI) [27] measures how much a person fears the symptoms of anxiety and was included to control for an individual’s propensity to be aware of and/or over-report symptoms. We determined migraine status at baseline using the Migraine ID questionnaire [28]. Physician diagnosis of asthma and hay fever were ascertained by self-report at baseline. Questionnaire return dates were used to categorize the season in which symptoms occurred as previously described [17]. We determined CRS_S_ status as previously reported [16]. Briefly, all available questionnaires up to and including the CT questionnaire were used to classify subjects as: current (met CRS_S_ criteria at time of CT), past (met criteria at prior timepoint but not at time of CT), or never (never met criteria). We included a binary indicator for whether the self-reported symptoms were ascertained from a questionnaire greater than 90 days from time of CT (if the CT symptom questionnaire was not completed), a duration consistent with CRS_S_ guidelines [1].

### Predictors of LCA group membership

To better understand the individuals assigned to these classes, we identified potential risk factors for class membership using latent class regression. We assessed four separate models using a standard (i.e. “one-step”) approach in which covariates can directly influence the makeup of the classes [29]. Model 1 included sex (female vs. male), ASI (z-transformed), self-reported physician diagnosis of hay fever (yes vs. no), age (z-transformed), and Charlson comorbidity index. Models 2 through 4 had the same base covariates as model 1 but further included one of self-reported physician diagnosed asthma, migraine status at baseline, or CRS_S_ status, respectively.

### Associations of LCA group membership with NSS

Most likely latent class membership was used as an indicator variable in subsequent analyses of overall NSS burden and individual symptoms. While this approach does not account for potential misclassification errors in membership assignment, the entropy of the final latent class model was high (0.86), suggesting low misclassification error. Therefore, the majority of individuals were assigned to the correct class. For modeling associations of LCA and selected covariates with overall NSS, we used least absolute deviation regression of the conditional median as it is more robust to non-normality of the dependent variable’s distribution [30, 31]. For associations with individual symptoms, we used multivariate (multiple outcome) ordered probit models [32–35] to allow for correlation among the related symptoms and was estimated by simulated maximum likelihood [33]. Ordered probit regression is similar to ordered logistic regression, however the former uses an inverse normal link function while the latter uses a logit link function [36]. Only four subjects reported nasal discharge in the highest frequency category (all of the time), so we combined that category with the one below (most of the time). Model building included fitting unadjusted models for a pool of potential risk factors selected *a priori* based on our prior work with CRS [10, 16, 17, 37–39]. Variables were selected for the final model if they were theoretically and/or statistically associated with NSS burden or were a demonstrated confounder of the association of latent class with NSS. Standard errors as well as bias-corrected and accelerated 95% confidence intervals were estimated via bootstrapping, with acceleration correcting for skewness in the bootstrap distribution [40]. We assessed potential influence of observations for each symptom’s unique model, from which three observations were deemed likely influential. As a sensitivity analysis, we assessed the final multivariate model with these observations removed, to better determine whether they had an impact on the final estimates.

EFA, LCA, and latent class regression were fit using Mplus v.8.1 (Muthén & Muthén, Los Angeles, CA) whereas all other models were fit using Stata v.15.1 (StataCorp, College Station, TX, USA).

## Results

### Overview of study sample

In our sample of 646 subjects, 18.6% (n = 120) had radiologic evidence of prior sinus surgery. Differences between those with and without evidence of surgery were observed in relation to proportion of females, self-reported physician diagnosed asthma, CRS_S_ status, and median LM and mLM scores (Table 1). Subjects with evidence of prior sinus surgery were excluded from subsequent analysis given the inability to determine whether prior sinus surgery affected observed sinus opacification and self-reported symptoms.

**Table 1:** Study sample characteristics comparing subjects with and without evidence of prior sinus surgery on sinus computed tomography.

Variables	Non-surgical (n = 526)		Surgical (n = 120)	
	Range	Median (IQR)	Range	Median (IQR)
Age at baseline (in years)	19.1–85.7	56.4 (17.3)	22.6–88.1	58.2 (15.3)
Body mass index (BMI; kg/m^2^)	17.5–59.3	30.0 (8.73)	15.7–51.2	30.9 (7.62)
Charlson comorbidity index^a^	0–7	2.00 (2.00)	0–7	2.00 (3.00)
Anxiety sensitivity index (0–64)^b^	0–64	12.0 (16.0)	0–52	13.0 (17.5)
Lund-Mackay (0–24)	0–22	0.00 (2.00)	0–22	3.00 (6.00)***
Modified Lund-Mackay (0–44)	0–39.5	1.50 (3.00)	0–42	4.50 (9.00)***
	**Column proportion (SE)**			
Female sex, n = 431	0.69 (0.02)		0.56 (0.05)**	
Non-white race/ethnicity, n = 26	0.05 (0.01)		0.00 (0.00)	
Medical Assistance (ever received)^c^, n = 56	0.09 (0.01)		0.08 (0.02)	
CRS_s_ status^d^				
Never, n = 73	0.13 (0.01)		0.04 (0.02)***	
Past, n = 249	0.39 (0.21)		0.37 (0.04)	
Current, n = 324	0.48 (0.02)		0.59 (0.04)*	
Self-reported physician diagnosis of asthma, n = 197	0.27 (0.02)		0.44 (0.05)***	
Self-reported physician diagnosed of hay fever, n = 361	0.55 (0.02)		0.61 (0.04)	
Migraine headache^e^, n = 229	0.36 (0.02)		0.33 (0.04)	

***p-value < 0.001,

**p-value < 0.01,

*p-value < 0.05;

p-values determined by Mann-Whitney-Wilcoxon U-test or Wald test.

CRSs, European Position Paper on Rhinosinusitis subjective symptoms definition for CRS classification; CT, computed tomography; EHR, electronic health record

^a^ Higher score indicates an individual has more chronic (e.g., coronary heart disease, chronic obstructive pulmonary disease) and chronic episodic (e.g., asthma, allergic rhinitis) disease diagnoses.

^b^ Higher score indicates greater sensitivity to physical symptoms of anxiety response.

^c^ Medical Assistance was determined from the EHR as a proxy for family socioeconomic status.

^d^ CRS status determined using self-reported symptoms relevant to CRS_s_ at all observed time-points up to and including closest to time of CT scan; never CRS = never met CRS_s_ criteria over follow-up; past CRS = met CRS_s_ criteria at some point in lifetime or over follow-up, but did not meet criteria at time of CT scan; current CRS = met CRS_s_ criteria at time of CT scan.

^e^ Based on responses to four questions, at baseline, from the ID Migraine questionnaire.

### LM vs. mLM scoring and nasal cavity opacification

Analysis of location-specific opacification scores using EFA showed both LM and mLM measure one underlying construct (S1 Table and S1 Fig). No meaningful differences with respect to factor composition were observed (i.e. all sinuses contributed to the underlying construct); however, frontal and sphenoid locations had larger factor loadings (i.e. more strongly associated with the underlying construct) when measured via LM (S2 Table). Results of EFA models were consistent when scores were categorized (S2 Table). Further, the addition of nasal cavity opacification did not alter factor compositions or interpretation of results (S2 Table).

### How should location specific scores be used?

We used LM scores for all subsequent analyses, given that EFA models showed no differences between mLM and LM, and LM is more commonly used. A three class LCA model had superior fit over the one and two class models (S3 Table). The classes were identifiable as “no/mild opacification, “localized opacification,” and “diffuse opacification” with prevalence estimates of 63.0%, 21.5%, and 15.5%, respectively (Table 2). Also, the probability of the most likely class being the true class (based on the estimated model) was: 97.4%, 94.7%, and 93.1% for the no/mild, localized, and diffuse class, respectively. Therefore, the majority of individuals were assigned to the correct class and misclassification error is low for this model. Descriptive characteristics of individuals assigned to each class showed substantial differences in median LM scores with the no/mild class having a score of 0, localized a score of 1, and diffuse a score of 7 (Table 2).

**Table 2:** Latent class posterior probabilities of sinus opacification and class membership characteristics for selected variables.

Sinus and OMC	Lund-Mackay sinus opacification score^a^ > 0 (overall %)	Sinus opacification probability		
		Class 1	Class 2	Class 3
OMC	11.6	0.40	11.6	56.8
Maxillary	38.8	6.60	100	85.0
Anterior ethmoid	23.6	6.70	22.2	93.9
Posterior ethmoid	14.1	3.50	0.00	76.5
Frontal	8.20	1.10	2.00	45.5
Sphenoid	7.20	3.90	0.00	30.5
Class prevalence (%)^b^		63.0%	21.5%	15.5%
Class name		No/mild opacification	Localized opacification	Diffuse opacification
Mean / median LM score (min, max)		0.18 / 0 (0, 4)	1.8 / 1 (1, 6)	7.2 / 7 (2, 22)
% LM ≥ 2		3%	44%	100%
% LM ≥ 3		0%	21%	94%
% LM ≥ 4		0%	9%	89%
% LM ≥ 5		0%	2%	78%
% female sex		74%	60%	54%
% migraine headache status^c^		37%	38%	30%
% self-reported physician diagnosis of hay fever		53%	59%	57%
% self-reported physician diagnosis of asthma		27%	24%	33%
% current CRS_s_^d^		49%	43%	54%
% past CRS_s_		39%	44%	33%
% any nasal cavity opacification (row %)		0%	0%	100%

Abbreviations: CRS_s_, European Position Paper on Rhinosinusitis subjective symptoms definition for CRS classification; LM, Lund-Mackay; OMC, osteomeatal complex

This table shows the proportion (%) of opacification in each sinus, given assignment to a particular class. Individuals in the diffuse class had higher LM scores, were somewhat more likely to have been diagnosed with asthma, and more likely to be male.

^a^ Based on CT scoring by two otorhinolaryngologists blinded to CRS_s_ status.

^b^ Based on estimated model.

^c^ Based on responses to four questions, at baseline, from the ID Migraine questionnaire.

^d^ CRS status determined using self-reported symptoms relevant to CRS_s_ at all observed time-points up to and including closest to time of CT scan; never CRS = never met CRS_s_ criteria over follow-up; past CRS = met CRS_s_ criteria at some point in lifetime or over follow-up, but did not meet criteria at time of CT scan; current CRS = met CRS_s_ criteria at time of CT scan.

### Risk factors for latent class membership

For all models, females (compared to males) had a 64–67% reduction in relative risk of being in the diffuse class (vs. no/mild) (S4 Table). There were also elevated relative risks for self-reported physician diagnosis of hay fever for localized and diffuse (vs. no/mild), self-reported physician diagnosis of asthma for diffuse (vs. no/mild), and migraine headache for localized (vs. no/mild); however, these did not reach statistical significance.

To make the use of our latent classes more readily applied in clinical settings, we developed simple criteria for assigning patients to these three groups based on sinus CT opacification (Table 3). For identifying individuals in the localized opacification class: maxillary sinus opacification alone, maxillary and anterior ethmoid opacification alone, or maxillary and OMC opacification alone had high sensitivity (98.5%), specificity (100%), and positive predictive value (100%) (Table 3). For identifying individuals in the diffuse opacification class: those not meeting criteria for the localized class and having opacification in at least two different sinus regions also had high sensitivity (100%), specificity (98.9%), and positive predictive value (94.3%) (Table 3).

**Table 3:** Diagnostic criteria for selection into localized or diffuse latent class.

Classification criteria	Latent Class
	Localized
Maxillary opacification alone **OR** maxillary and anterior ethmoid opacification alone **OR** maxillary and OMC opacification alone	Sensitivity: 98.5% (131 / 133)
	Specificity: 100% (393 / 393)
	Positive predictive value: 100% (131 / 131)
	Negative predictive value: 99.5% (393 / 395)
	**Diffuse**
Does not meet criteria for localized class **AND** opacification in at least 2 different sinus regions	Sensitivity: 100% (83 / 83)
	Specificity: 98.9% (438 / 443)
	Positive predictive value: 94.3% (83 / 88)
	Negative predictive value: 100% (438 / 438)

Abbreviations: OMC, osteomeatal complex

This table describes simple, logical rules that can be used by researchers and clinicians to assign individuals to one of the latent classes described in this study, without the need for using latent class analysis. For example, in our analysis, 100% of individuals with maxillary sinus opacification alone, maxillary and anterior ethmoid opacification alone, or maxillary and OMC opacification together, were assigned to the localized class.

### Latent class membership informing LM score cutoff selection

**Fig 1:**
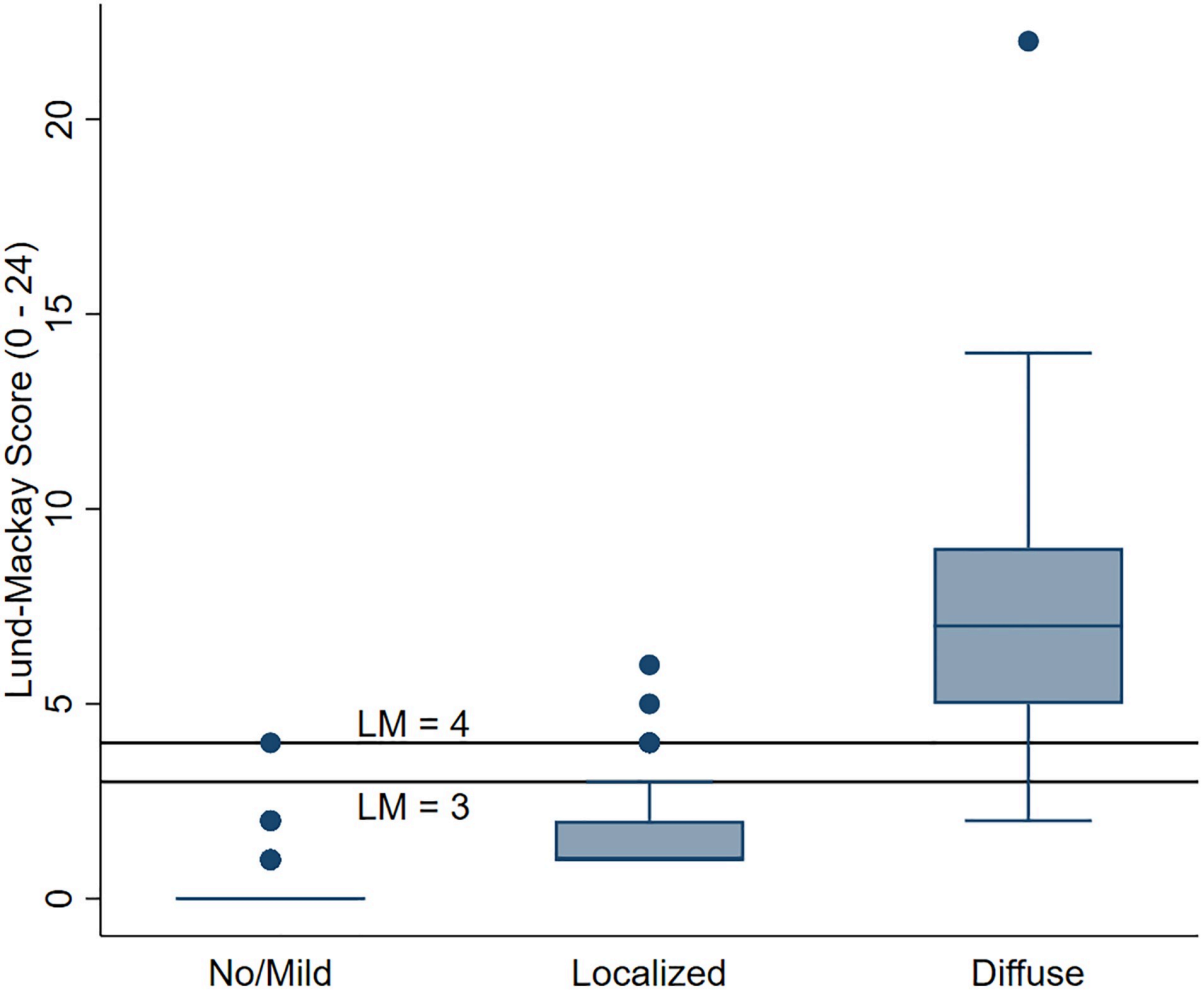
Lund-Mackay distributions within latent classes, comparing two LM cutoffs (LM ≥ 4 and ≥ 3). Cutoffs are marked with horizontal black lines.

We compared the distributions of LM scores in each class against LM cutoffs of ≥ 4 and ≥ 3 (Fig 1). LM ≥ 4 tended to exclude individuals in the diffuse class more severely than LM ≥ 3. While LM ≥ 3 still excluded individuals in the diffuse class (n = 5), it provided a balance of including subjects from the diffuse class (94%) while also excluding 99.7% of the no/mild class and 79% of localized.

### Associations of latent class with overall symptom burden and core CRS_S_ symptoms

The distribution of total NSS burden scores was shifted to higher values for the diffuse class (S2 Fig). In adjusted analysis, diffuse opacification (vs. no/mild) was associated with more NSS, with a median index value increase of 1.15 (95% confidence interval [CI]: 0.29, 2.02) (S5 Table).

For individual symptoms, diffuse opacification was positively associated with nasal blockage (β = 0.27; 95% CI: 0.01, 0.53) and smell loss (β = 0.37; 95% CI: 0.10, 0.63) (S6 Table), conferring increased probability of reporting symptoms (S3 Fig). Migraine headache modified associations of localized latent class membership with nasal discharge and post-nasal drip (S6 Table); among individuals in the localized class, those with migraine headache were less likely to report frequent (i.e., most or all of the time in the past three months) nasal discharge and post-nasal drip, compared to similar individuals without migraine headache (S4 Fig). In a sensitivity analysis, three potentially influential observations were removed prior to model estimation; effect estimates were not substantively changed (S7 Table).

## Discussion

Improving and standardizing measurement of sinus opacification is critical to advancing research in CRS as inconsistent or weakly-justified approaches to characterizing sinus opacification makes inferences across studies difficult. To the best of our knowledge, this is first study to evaluate LM vs. mLM, evaluate whether nasal cavity opacification should be included in these scoring approaches, and identify other ways to classify location-specific scores, in a general population representative sample.

LM has been recommended by the Task Force on Rhinosinusitis due to its simplicity and ease in interpretation [5]. Inability to assess progression of disease prompted the development of modified scoring approaches including the mLM system [8]. No prior studies had compared these two approaches in a general population sample of persons without prior surgery and representing a broad spectrum of disease. Since mLM and LM both measured a single dimension of sinus disease, we found no advantage of using mLM over LM for research. Also, neither approach includes nasal cavity opacification as part of its scoring, but opacification in the nasal cavity might be indicative of nasal polyposis, an important CRS phenotype (CRSwNP) [1]. While we found no evidence to support inclusion of nasal cavity opacification, we had relatively few persons with any nasal cavity opacification in our sample (5.3% of the original 646 sample).

LCA identified three subgroups of sinus opacification: no or mild, isolated opacification; localized, mainly maxillary opacification; and diffuse opacification, almost always including the anterior ethmoid. Therefore, current approaches to summing scores across all sinus locations may hide clinically useful information about the location and pattern of opacification. Despite prior studies that suggested that women had a higher risk of CRS [16, 41–43], we found an interesting difference by sex in the extent of sinus opacification. Male sex has previously been associated with higher LM scores [15], and was supported by our latent classes in which men were over 2.7 times more likely to be in the diffuse (vs. no/mild) class than women. Hay fever and asthma both trended towards an association with the diffuse class, both of which have previously been associated with the occurrence of diagnostic codes for CRS [37, 44], CRS symptoms (CRS_S_) [16, 45], and sinus opacification on CT imaging [15, 46]. Future studies should explore whether latent class membership is associated with CRS endotypes and response to treatment as these classes could have relevance to disease management.

These subgroups suggested that a different approach to the use of location-specific sinus opacification may be advantageous. The current suggested guideline for objective evidence indicative of CRS is an LM cutoff of LM ≥ 4 [6], however there are several limitations with the study from which that guideline was established. That study used CT scans from subjects with indications requiring CT imaging, therefore they do not necessarily represent the general population. Further, individuals with suspected or confirmed CRS were excluded from the analysis, thereby making the distribution of LM scores in the target sample unavailable. However, if the standard approach to a single LM score cutoff is to be used, a cutoff of LM ≥ 3 may be more appropriate, given its greater inclusion of individuals from the diffuse opacification class, which is taken to represent the group of individuals with the most sinus disease.

Lastly, we addressed the oft-cited lack of correlation between objective and subjective evidences of CRS. In adjusted analysis, diffuse latent class membership was associated with overall NSS burden; however, this overall association seemed to be primarily driven by associations with nasal blockage and smell loss. This had never been evaluated in a general population representative sample across a broad spectrum of disease and in the appropriate causal direction. Though, a study using CT scans from subjects presenting with CRS symptoms at an otorhinolaryngology care clinic found that subjects with LM ≥ 4 were more likely to report smell loss [47]. A similar finding was observed among subjects with non-CRS related indications requiring sinus CT imaging, in which LM ≥ 4 was associated with more nasal blockage and smell loss [15]. These findings are perhaps not surprising, as one of the potential causes of olfactory dysfunction is the blockade of air flow to the olfactory zone.

Our study had several strengths. We used a population-based sample representing a broad spectrum of sinus disease, including non-surgical patients which are typically excluded from CT-based studies of CRS. Additionally, we had a large sample size and used a rigorous and novel approach to our analysis. This study, however, is not without limitations. Due to model identifiability, we were unable to assess LCA models beyond sinus opacification patterns based on presence/absence of opacification (i.e., LM score = 0 or > 0. Thus, we could not identify subgroups which also described severity of disease (e.g., LM score of 0, 1, or 2). Further, we only have a cross-sectional measurement of sinus CT opacification and as such cannot assess longitudinal changes in opacification over time, which was a primary motivation for the development of the mLM scoring approach [8]. Future studies should assess whether scoring approaches differ in ability to detect changes in opacification over time and to evaluate the natural history of the identified latent classes.

## Conclusions

- There were no differences between mLM and LM scoring approaches.
- There was no benefit of including nasal cavity opacification in scoring approaches.
- Using LCA, three sinus opacification subgroups could be identified.
- Males more likely to be categorized in the diffuse sinus disease class.
- Persons categorized in the diffuse sinus opacification class had more nasal and sinus symptoms.

## Supporting information

S1 Fig Example scree plot for exploratory factor analysis models. Scree plot from modified Lund-Mackay scored locations in the raw (uncategorized) scale and no nasal cavity included. All scree plots assessed were similar to the one shown above. Larger eigenvalues indicate greater variance explained by the associated factor. Given the large drop in variability explained with additional factors, this plot suggest that a single factor is appropriate for the exploratory factor analysis model.
(TIF)

S2 Fig Box-and-whisker plot of CRS_s_ symptom index within latent classes. The symptom index was created by summing frequency scores (0 to 4 from never to all the time) for four CRS symptom groups. See Methods for additional details. Nasal and sinus symptoms were more frequent and more severe in the diffuse latent class compared to the other classes, as indicated by a greater median symptom index score.
(TIF)

S3 Fig Marginal probabilities of self-reported symptoms at all frequency categories (in the past three months), by latent class. Estimates based on an adjusted multivariate ordered probit regression model. Nasal blockage (A), smell loss (B), facial pain (C), and facial pressure (D). Frequency categories were: never, once in a while (“once”), some of the time (“some”), most of the time (“most”), and all of the time (“all”). Individuals in the diffuse class were more likely to report nasal blockage and smell loss, compared to those in the other classes.
(TIF)

S4 Fig Marginal probabilities of self-reported symptoms at all frequency categories (in the past three months), by latent class and migraine status. Estimates based on an adjusted multivariate ordered probit regression model. Nasal discharge (A) and post-nasal drip (B). Frequency categories were: never, once in a while (“once”), some of the time (“some”), most of the time (“most”), and all of the time (“all”). The two highest frequency categories were combined for nasal discharge since there were only four observations in the highest category. Individuals in the localized opacification class who reported having migraine headaches were less likely to report nasal discharge and post-nasal drip, compared to those who did not report migraine headaches.
(TIF)

S1 Table Fit of exploratory factor analysis models of modified Lund-Mackay (mLM) and Lund-Mackay (LM) scored sinuses. Sinuses scored on original and categorized (reduced) scales, with and without addition of binary (none vs. at least a score of one) nasal cavity opacification.
(DOCX)

S2 Table One factor exploratory factor analysis models of modified Lund-Mackay (mLM) and Lund-Mackay (LM) scored sinuses. Sinuses scored on original and categorized (reduced) scales, with and without addition of binary (none vs. at least a score of one) nasal cavity opacification.
(DOCX)

S3 Table Lund-Mackay sinus opacification patterns and latent class analyses fits (one to three classes). (DOCX)

S4 Table Unadjusted and adjusted associations of selected variables with latent class membership^a^. (DOCX)

S5 Table Associations of selected variables with CRS_s_ symptom index^a^ at the median (0.50 quantile). (DOCX)

S6 Table Associations of selected variables with six core CRS_s_ symptoms in multivariate (multiple-outcome) ordered probit^a^ analysis. (DOCX)

S7 Table Associations of six core CRS_s_ symptoms with selected covariates in a multivariate (multiple-outcome) ordered probit^a^ model with three influential observations removed. (DOCX)

S1 Data Anonymized data set for replication of findings. (CSV)

S2 Data Description of variables included in anonymized data set. (XLSX)
