# Supplementary figures and images for: A new approach to categorization of radiologic inflammation in chronic rhinosinusitis
Source: PLoS One. 2020 Jun 29;15(6):e0235432. doi: 10.1371/journal.pone.0235432 (PMC7323942; doi:10.1371/journal.pone.0235432)

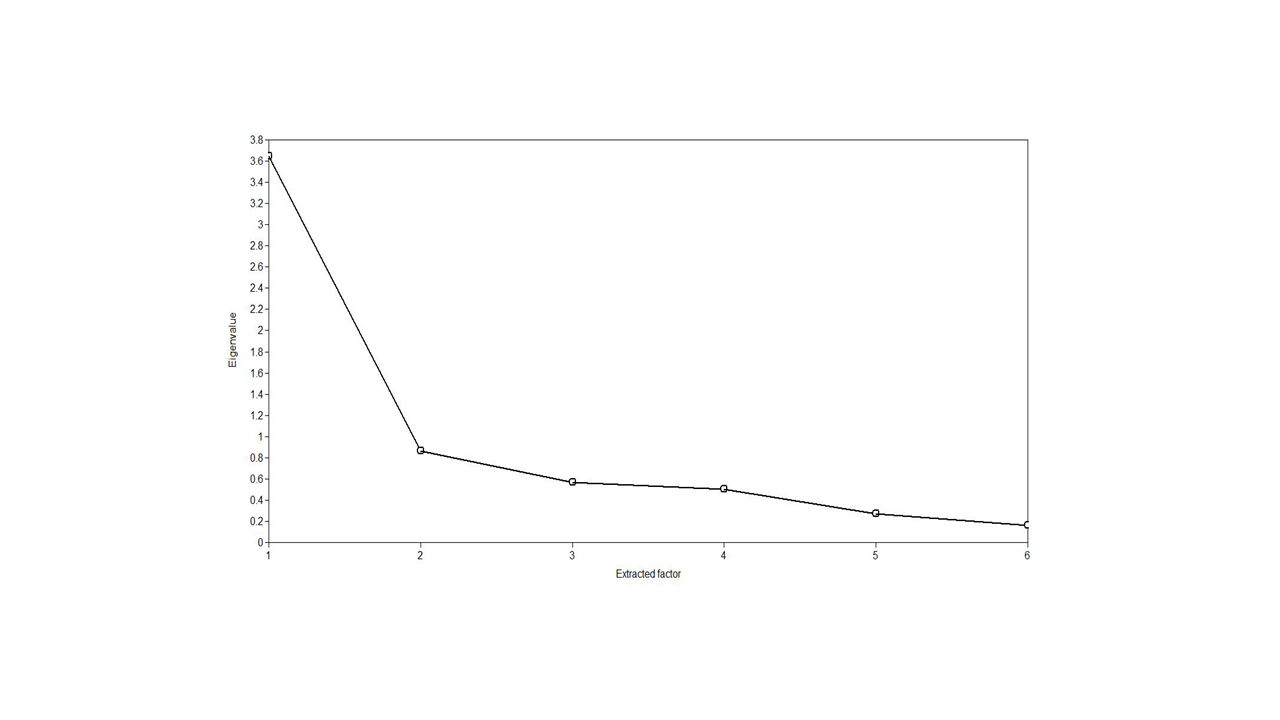

Supplement: S1 Fig — Scree plot from modified Lund-Mackay scored locations in the raw (uncategorized) scale and no nasal cavity included. All scree plots assessed were similar to the one shown above. Larger eigenvalues indicate greater variance explained by the associated factor. Given the large drop in variability explained with additional factors, this plot suggest that a single factor is appropriate for the exploratory factor analysis model. (TIF) [file pone.0235432.s001.tif]

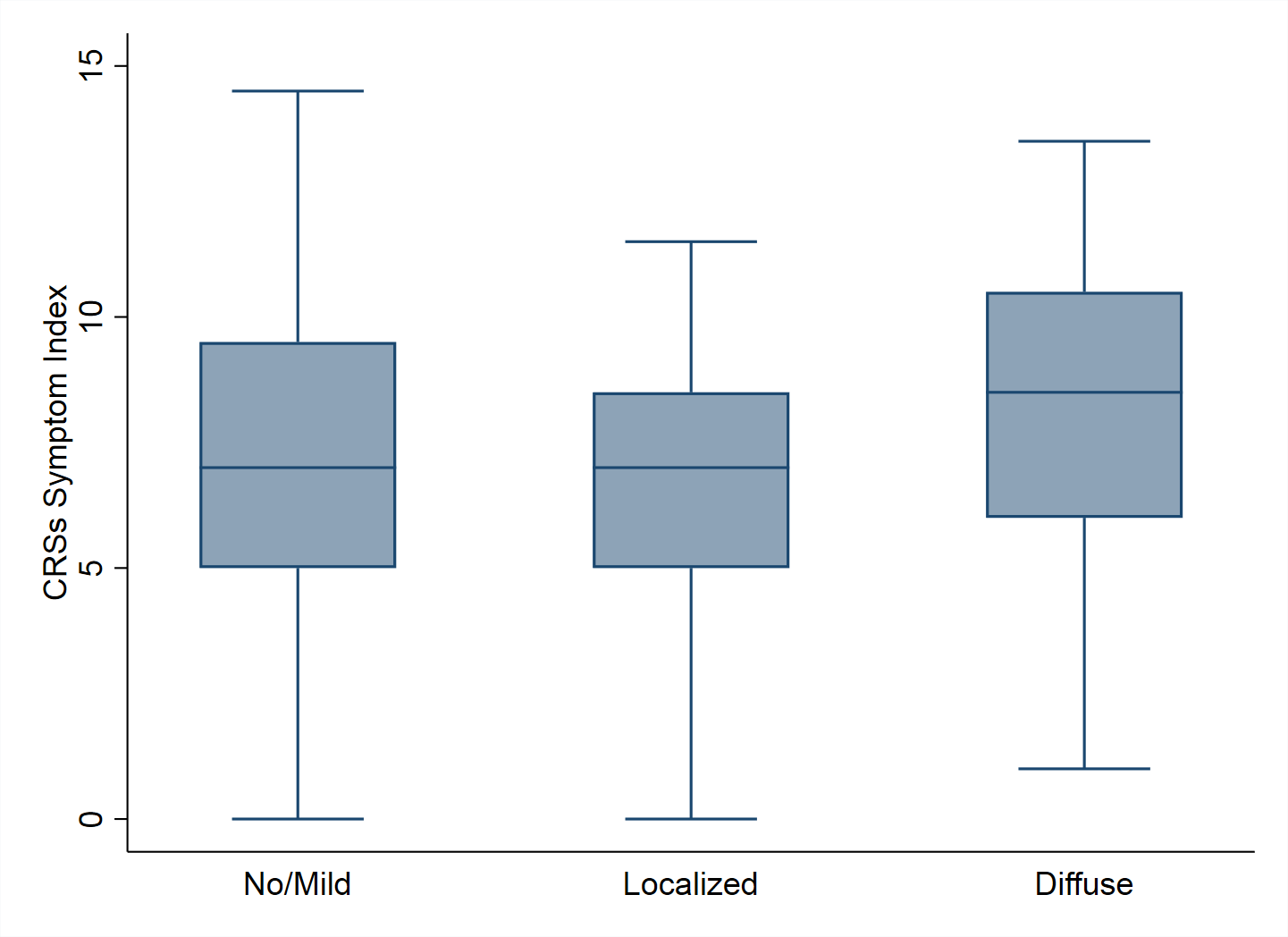

Supplement: S2 Fig — The symptom index was created by summing frequency scores (0 to 4 from never to all the time) for four CRS symptom groups. See Methods for additional details. Nasal and sinus symptoms were more frequent and more severe in the diffuse latent class compared to the other classes, as indicated by a greater median symptom index score. (TIF) [file pone.0235432.s002.tif]

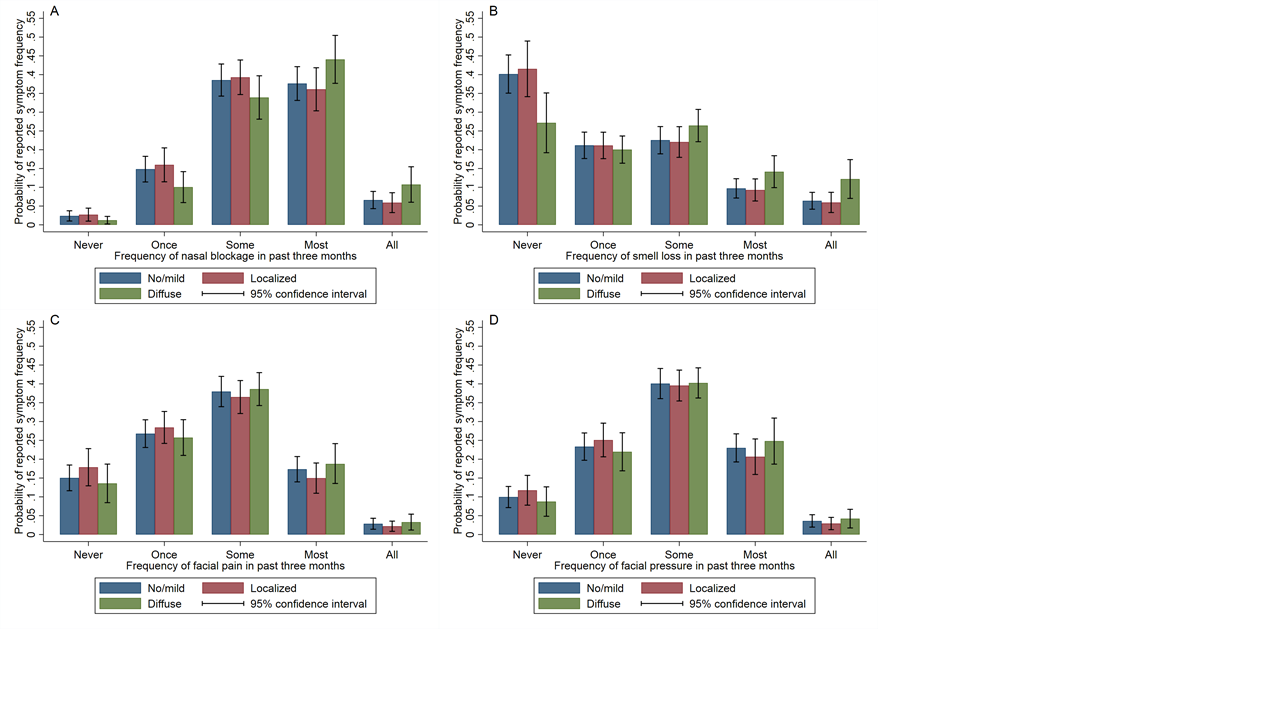

Supplement: S3 Fig — Estimates based on an adjusted multivariate ordered probit regression model. Nasal blockage (A), smell loss (B), facial pain (C), and facial pressure (D). Frequency categories were: never, once in a while (“once”), some of the time (“some”), most of the time (“most”), and all of the time (“all”). Individuals in the diffuse class were more likely to report nasal blockage and smell loss, compared to those in the other classes. (TIF) [file pone.0235432.s003.tif]

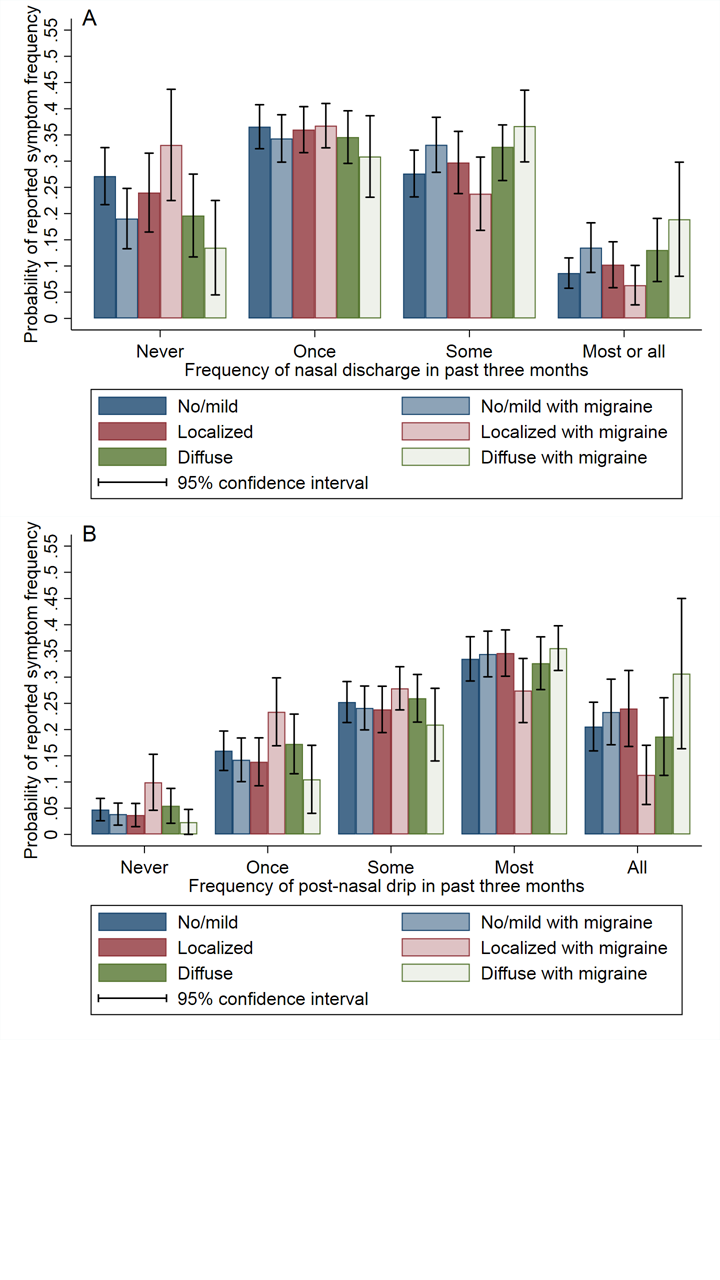

Supplement: S4 Fig — Estimates based on an adjusted multivariate ordered probit regression model. Nasal discharge (A) and post-nasal drip (B). Frequency categories were: never, once in a while (“once”), some of the time (“some”), most of the time (“most”), and all of the time (“all”). The two highest frequency categories were combined for nasal discharge since there were only four observations in the highest category. Individuals in the localized opacification class who reported having migraine headaches were less likely to report nasal discharge and post-nasal drip, compared to those who did not report migraine headaches. (TIF) [file pone.0235432.s004.tif]
