# Supplementary material for: A new approach to categorization of radiologic inflammation in chronic rhinosinusitis
Source: PLoS One. 2020 Jun 29;15(6):e0235432. doi: 10.1371/journal.pone.0235432 (PMC7323942; doi:10.1371/journal.pone.0235432)
Supplement: S1 Table — Sinuses scored on original and categorized (reduced) scales, with and without addition of binary (none vs. at least a score of one) nasal cavity opacification. (DOCX) [file pone.0235432.s005.docx]

**S1 Table.** **Fit of exploratory factor analysis models of modified Lund-Mackay (mLM) and Lund-Mackay (LM) scored sinuses.** Sinuses score on original and categorized (reduced) scales, with and without addition of binary (none vs. at least a score of one) nasal cavity opacification.

|  | **No nasal cavity included** | | | **Nasal cavity included** | |
| --- | --- | --- | --- | --- | --- |
|  | **Original scale** | | **Categorized scale** | **Original scale** | **Categorized scale** |
| **mLM scale** |  |  | |  |  |
| SSABIC |  |  | |  |  |
| 1 factor | 6730.4 | 4342.3 | | 6969.1 | 4407.6 |
| 2 factor | - | 4322.4 | | - | 4390.0 |
| Eg > 1.0 | 1 | 1 | | 1 | 1 |
| **LM scale** |  |  | |  |  |
| SSABIC |  |  | |  |  |
| 1 factor | 2890.9 | 2196.4 | | 2940.3 | 2240.7 |
| 2 factor | - | 2192.7 | | 2929.9 | 2238.2 |
| Eg > 1.0 | 1 | 1 | | 1 | 1 |
| Abbreviations: Eg = eigenvalue; LM = Lund-Mackay; mLM = modified Lund-Mackay; SSABIC = sample size-adjusted Bayesian information criterion | | | | | |
