# Supplementary material for: A new approach to categorization of radiologic inflammation in chronic rhinosinusitis
Source: PLoS One. 2020 Jun 29;15(6):e0235432. doi: 10.1371/journal.pone.0235432 (PMC7323942; doi:10.1371/journal.pone.0235432)
Supplement: S2 Table — Sinuses scored on original and categorized (reduced) scales, with and without addition of binary (none vs. at least a score of one) nasal cavity opacification. (DOCX) [file pone.0235432.s006.docx]

**S2 Table.** **One factor exploratory factor analysis models of modified Lund-Mackay (mLM) and Lund-Mackay (LM) scored sinuses**. Sinuses scored on original and categorized (reduced) scales, with and without addition of binary (none vs. at least a score of one) nasal cavity opacification.

| **OMC and sinus locations** | **No nasal cavity included** | | | | **Nasal cavity included** | | | |
| --- | --- | --- | --- | --- | --- | --- | --- | --- |
|  | **Original scale** | | **Categorized scale** | | **Original scale** | | **Categorized scale** | |
|  | **Loadings** | **Communalities** | **Loadings** | **Communalities** | **Loadings** | **Communalities** | **Loadings** | **Communalities** |
| **mLM**  OMC | 0.80 | 0.63 | 0.80 | 0.65 | 0.89 | 0.80 | 0.80 | 0.65 |
| Maxillary | 0.67 | 0.45 | 0.64 | 0.41 | 0.75 | 0.57 | 0.64 | 0.41 |
| Anterior ethmoid | 0.94 | 0.89 | 1.00 | 1.00 | 0.97 | 0.94 | 1.00 | 1.00 |
| Posterior ethmoid | 0.81 | 0.66 | 0.83 | 0.69 | 0.86 | 0.74 | 0.83 | 0.69 |
| Frontal | 0.71 | 0.51 | 0.75 | 0.56 | 0.77 | 0.60 | 0.75 | 0.56 |
| Sphenoid | 0.34 | 0.12 | 0.47 | 0.22 | 0.40 | 0.16 | 0.47 | 0.22 |
| Nasal cavity |  |  |  |  | 0.66 | 0.44 | 0.78 | 0.60 |
| **LM**  OMC | 0.85 | 0.72 | 0.84 | 0.71 | 0.84 | 0.71 | 0.83 | 0.70 |
| Maxillary | 0.72 | 0.52 | 0.65 | 0.42 | 0.72 | 0.52 | 0.66 | 0.44 |
| Anterior ethmoid | 0.90 | 0.81 | 0.92 | 0.85 | 0.90 | 0.81 | 0.92 | 0.84 |
| Posterior ethmoid | 0.91 | 0.83 | 0.85 | 0.72 | 0.91 | 0.82 | 0.85 | 0.72 |
| Frontal | 0.85 | 0.73 | 0.86 | 0.74 | 0.85 | 0.73 | 0.85 | 0.72 |
| Sphenoid | 0.61 | 0.37 | 0.61 | 0.37 | 0.62 | 0.39 | 0.61 | 0.37 |
| Nasal cavity |  |  |  |  | 0.87 | 0.75 | 0.93 | 0.86 |
| Abbreviations: LM = Lund-Mackay; mLM = modified Lund-Mackay; OMC = osteomeatal complex | | | | | | | | |
