# Supplementary material for: A new approach to categorization of radiologic inflammation in chronic rhinosinusitis
Source: PLoS One. 2020 Jun 29;15(6):e0235432. doi: 10.1371/journal.pone.0235432 (PMC7323942; doi:10.1371/journal.pone.0235432)
Supplement: S3 Table — (DOCX) [file pone.0235432.s007.docx]

| **Lund-Mackay sinus opacification score (in order): OMC, maxillary (M), anterior ethmoid (AE), posterior ethmoid (PE), frontal (F), and sphenoid (S): None (0) or at least 1 (1)** |  | | | | | |  | **Expected pattern frequency (standardized residual)** | | |
| --- | --- | --- | --- | --- | --- | --- | --- | --- | --- | --- |
|  | **OMC** | **M** | **AE** | **PE** | **F** | **S** | **Observed, n** | **1-class model** | **2-class model** | **3-class model** |
| 8 most frequently observed patterns (84.8% of subjects) | 0 | 0 | 0 | 0 | 0 | 0 | 265 | 159.3 (10.0) | 253.7 (0.99) | 264 (0.09) |
|  | 0 | 1 | 0 | 0 | 0 | 0 | 94 | 100.9 (-0.76) | 104.8 (-1.18) | 94.7 (-0.08) |
|  | 0 | 1 | 1 | 0 | 0 | 0 | 26 | 31.1 (-0.95) | 14.88 (2.92) | 26 (0.10) |
|  | 0 | 0 | 1 | 0 | 0 | 0 | 19 | 49.1 (-4.51) | 27.3 (-1.63) | 19 (-0.09) |
|  | 1 | 1 | 1 | 1 | 0 | 0 | 12 | 0.67 (13.87) | 11.3 (0.20) | 10.7 (0.39) |
|  | 0 | 0 | 0 | 0 | 0 | 1 | 11 | 12.4 (-0.40) | 8.06 (1.04) | 10.8 (0.05) |
|  | 1 | 1 | 0 | 0 | 0 | 0 | 11 | 13.2 (-0.62) | 3.44 (4.09) | 10.3 (0.23) |
|  | 1 | 1 | 1 | 1 | 1 | 1 | 8 | 0.0 (117.5) | 3.45 (2.46) | 3.94 (2.05) |
| Latent class model fit statistics | | | | | | |  |  |  |  |
| Entropy | | | | | | |  | NA | 0.91 | 0.86 |
| AIC | | | | | | |  | 2664.4 | 2196 | 2172.8 |
| BIC | | | | | | |  | 2690.0 | 2251.4 | 2258.1 |
| SSABIC | | | | | | |  | 2671.0 | 2210.1 | 2194.6 |
| Pearson chi-square | | | | | | |  | 1955.2 (p < 0.0001) | 75.9 (p = 0.01) | 36.8 (p = 0.73) |
| Vuong-Lo-Mendell-Rubin LRT | | | | | | |  | NA | 482.5 (p < 0.0001) | 36.4 (p < 0.0001) |
| Bootstrapped LRT | | | | | | |  | NA | 482.5 (p < 0.0001) | 37.2 (p< 0.0001) |
| Latent class prevalence (estimated from model) | | | | | | |  |  |  |  |
| Class 1 | | | | | | |  | 100% | 83.5% | 63.0% |
| Class 2 | | | | | | |  |  | 16.5% | 21.5% |
| Class 3 | | | | | | |  |  |  | 15.5% |
| Abbreviations: AIC = Akaike’s information criterion; BIC = Bayesian information criterion; SSABIC = sample size-adjusted Bayesian information criterion; LRT = likelihood ratio test; OMC = osteomeatal complex | | | | | | | | | | |

**S3 Table.** **Lund-Mackay sinus opacification patterns and latent class analyses fits (one to three classes).**
