# Supplementary material for: A new approach to categorization of radiologic inflammation in chronic rhinosinusitis
Source: PLoS One. 2020 Jun 29;15(6):e0235432. doi: 10.1371/journal.pone.0235432 (PMC7323942; doi:10.1371/journal.pone.0235432)
Supplement: S4 Table — (DOCX) [file pone.0235432.s008.docx]

**S4 Table.** **Unadjusted and adjusted associations of selected variables with latent class membership^a^.**

| **Variables** | **No/mild** | **Relative risk ratios (RRR) and 95% confidence intervals** | | | |
| --- | --- | --- | --- | --- | --- |
|  |  | **Localized** | | **Diffuse** | |
|  |  | **Unadjusted** | **Adjusted** | **Unadjusted** | **Adjusted** |
| **Model 1** |  |  |  |  |  |
| Female sex (vs. male) | REF | 0.58 (0.23, 1.46) | 0.55 (0.19, 1.64) | 0.37 (0.21, 0.63)*** | 0.34 (0.19, 0.60)*** |
| Anxiety sensitivity index (z-transformed) | REF | 0.89 (0.70, 1.11) | 0.89 (0.65, 1.22) | 0.97 (0.77, 1.23) | 0.98 (0.77, 1.25) |
| Self-reported physician diagnosis of hay fever (vs. no) | REF | 1.36 (0.80, 2.31) | 1.64 (0.82, 3.29) | 1.28 (0.75, 2.18) | 1.49 (0.86, 2.57) |
| Age (years; z-transformed) | REF | 1.06 (0.80, 1.40) | 1.05 (0.71, 1.55) | 0.94 (0.73, 1.22) | 0.91 (0.69, 1.20) |
| Charlson comorbidity index (z-transformed) | REF | 0.93 (0.71, 1.21) | 0.91 (0.64, 1.29) | 0.88 (0.67, 1.17) | 0.91 (0.67, 1.23) |
| **Model 2** |  |  |  |  |  |
| Female sex (vs. male) | REF | 0.58 (0.23, 1.46) | 0.63 (0.38, 1.07) | 0.37 (0.21, 0.63)*** | 0.34 (0.20, 0.60)*** |
| Anxiety sensitivity index (z-transformed) | REF | 0.89 (0.70, 1.11) | 0.90 (0.71, 1.13) | 0.97 (0.77, 1.23) | 0.97 (0.76, 1.25) |
| Self-reported physician diagnosis of hay fever (vs. no) | REF | 1.36 (0.80, 2.31) | 1.59 (1.00, 2.53) | 1.28 (0.75, 2.18) | 1.40 (0.80, 2.46) |
| Age (years; z-transformed) | REF | 1.06 (0.80, 1.40) | 1.02 (0.80, 1.29) | 0.94 (0.73, 1.22) | 0.92 (0.70, 1.21) |
| Charlson comorbidity index (z-transformed) | REF | 0.93 (0.71, 1.21) | 0.98 (0.75, 1.26) | 0.88 (0.67, 1.17) | 0.86 (0.64, 1.15) |
| Self-reported physician diagnosis of asthma (vs. no) | REF | 0.79 (0.48, 1.31) | 0.75 (0.42, 1.32) | 1.31 (0.75, 2.28) | 1.45 (0.79, 2.65) |
| **Model 3** |  |  |  |  |  |
| Female sex (vs. male) | REF | 0.58 (0.23, 1.46) | 0.50 (0.06, 4.06) | 0.37 (0.21, 0.63)*** | 0.36 (0.20, 0.64)*** |
| Anxiety sensitivity index (z-transformed) | REF | 0.89 (0.70, 1.11) | 0.89 (0.57, 1.41) | 0.97 (0.77, 1.23) | 1.00 (0.77, 1.29) |
| Self-reported physician diagnosis of hay fever (vs. no) | REF | 1.36 (0.80, 2.31) | 1.62 (0.71, 3.72) | 1.28 (0.75, 2.18) | 1.49 (0.86, 2.59) |
| Age (years; z-transformed) | REF | 1.06 (0.80, 1.40) | 1.11 (0.52, 2.35) | 0.94 (0.73, 1.22) | 0.88 (0.66, 1.16) |
| Charlson comorbidity index (z-transformed) | REF | 0.93 (0.71, 1.21) | 0.89 (0.53, 1.51) | 0.88 (0.67, 1.17) | 0.91 (0.67, 1.24) |
| Migraine headache status (vs. no)^b^ | REF | 1.13 (0.68, 1.89) | 1.38 (0.50, 3.79) | 0.65 (0.36, 1.18) | 0.72 (0.37, 1.40) |
| **Model 4** |  |  |  |  |  |
| Female sex (vs. male) | REF | 0.58 (0.23, 1.46) | 0.52 (0.11, 2.46) | 0.37 (0.21, 0.63)*** | 0.33 (0.19, 0.59)*** |
| Anxiety sensitivity index (z-transformed) | REF | 0.89 (0.70, 1.11) | 0.92 (0.61, 1.38) | 0.97 (0.77, 1.23) | 0.96 (0.75, 1.23) |
| Self-reported physician diagnosis of hay fever (vs. no) | REF | 1.36 (0.80, 2.31) | 1.73 (0.69, 4.34) | 1.28 (0.75, 2.18) | 1.48 (0.84, 2.60) |
| Age (years; z-transformed) | REF | 1.06 (0.80, 1.40) | 1.07 (0.68, 1.70) | 0.94 (0.73, 1.22) | 0.90 (0.68, 1.19) |
| Charlson comorbidity index (z-transformed) | REF | 0.93 (0.71, 1.21) | 0.90 (0.61, 1.34) | 0.88 (0.67, 1.17) | 0.90 (0.67, 1.22) |
| CRS_s_ status (vs. never CRS_s_)^c^  Past CRS_s_  Current CRS_s_ | REF | 1.11 (0.50, 2.46) 0.82 (0.38, 1.81) | 1.10 (0.36, 3.33) 0.84 (0.32, 2.23) | 0.76 (0.33, 1.74) 1.02 (0.47, 2.22) | 0.73 (0.30, 1.74) 1.03 (0.45, 2.38) |
| ***p-value < 0.001, **p-value < 0.01, *p-value < 0.05  Abbreviations: CRS_s_ = European Position Paper on Rhinosinusitis subjective symptoms definition for CRS classification  ^a^ Did not fix measurement error associated with latent class membership allowing covariates to influence makeup of latent classes.  ^b^ Based on responses to four questions, at baseline, from the ID Migraine questionnaire.  ^c^ CRS status determined using self-reported symptoms relevant to CRS_s_ at all observed time-points up to and including closest to time of CT scan; never CRS = never met CRS_s_ criteria over follow-up; past CRS = met CRS_s_ criteria at some point in lifetime or over follow-up, but did not meet criteria at time of CT scan; current CRS = met CRS_s_ criteria at time of CT scan. | | | | | |
