# Supplementary material for: A new approach to categorization of radiologic inflammation in chronic rhinosinusitis
Source: PLoS One. 2020 Jun 29;15(6):e0235432. doi: 10.1371/journal.pone.0235432 (PMC7323942; doi:10.1371/journal.pone.0235432)
Supplement: S5 Table — (DOCX) [file pone.0235432.s009.docx]

**S5 Table.** **Associations of selected variables with CRS_s_ symptom index^a^ at the median (0.50 quantile).**

| **Variables** | **β-coefficient (BCa^b^ confidence interval)** |
| --- | --- |
| Latent class (vs. no/mild)  Localized  Diffuse | 0.01 (-0.74, 0.61) 1.15 (0.29, 2.02)* |
| Female sex (vs. male) | -0.24 (-1.06, 0.31) |
| Anxiety sensitivity index (z-transformed) | 0.55 (0.22, 0.94)* |
| Migraine headache status (vs. no)^c^ | 1.10 (0.56, 1.82)* |
| Self-reported physician diagnosis of hay fever (vs. no) | 0.50 (-0.12, 1.12) |
| Season questionnaire returned (vs. fall)^d^  Winter  Spring  Summer | 0.50 (-0.42, 1.35) 0.54 (-0.24, 1.30) 0.18 (-0.63, 1.22) |
| * Crossed inferential boundary (evidenced by confidence interval not crossing 0.00); model additionally adjusted for smoking status (former or current vs. never) at baseline and binary indicator for whether symptoms taken from questionnaire occurred > 90 days from time of CT scan.  Abbreviations: BCa = bias-corrected and accelerated; CRS_s_ = European Position Paper on Rhinosinusitis subjective symptoms definition for CRS classification;  ^a^ Sum of 4 core CRS_s_ symptom groups (nasal blockage; nasal discharge and post-nasal drip; smell loss; facial pain and facial pressure) self-reported at questionnaire closest to time of CT scan.  ^b^ 95% bias-corrected and accelerated confidence interval based on bootstrap estimation; adjusted for skew of bootstrap distribution.  ^c^ Based on responses to four questions, at baseline, from the ID Migraine questionnaire.  ^d^ Fall = 22 September to 21 December; winter = 22 December to 21 March; spring = 22 March to 22 June; summer = 22 June to 21 September. | |
