# Supplementary material for: A new approach to categorization of radiologic inflammation in chronic rhinosinusitis
Source: PLoS One. 2020 Jun 29;15(6):e0235432. doi: 10.1371/journal.pone.0235432 (PMC7323942; doi:10.1371/journal.pone.0235432)
Supplement: S7 Table — (DOCX) [file pone.0235432.s011.docx]

**S7 Table.** **Associations of six core CRS_s_ symptoms with selected covariates in a multivariate (multiple-outcome) ordered probit^a^ model with three influential observations removed.**

| **Variables** | **Nasal blockage** | **Nasal discharge^b^** | **Post-nasal drip^c^** | **Smell loss** | **Facial pain^d^** | **Facial pressure^e^** |
| --- | --- | --- | --- | --- | --- | --- |
| Latent class (vs. no/mild)  Localized  Diffuse | -0.06 (-0.28, 0.16) 0.27 (0.01, 0.53)* | 0.11 (-0.17, 0.38) 0.22 (-0.09, 0.53) | 0.12 (-0.15, 0.39) -0.07 (-0.38, 0.24) | -0.04 (-0.26, 0.19) 0.37 (0.10, 0.63)** | -0.13 (-0.35, 0.09) 0.07 (-0.19, 0.33) | -0.10 (-0.32, 0.11) 0.08 (-0.18, 0.34) |
| Migraine headache status (vs. no)^f^ | 0.23 (0.03, 0.43)* | 0.27 (0.02, 0.52)* | 0.10 (-0.15, 0.35) | 0.30 (0.10, 0.50)** | 0.49 (0.29, 0.70)*** | 0.46 (0.25, 0.66)*** |
| Latent class by migraine headache status interaction  Localized  Diffuse |  | -0.56 (-1.00, -0.14)* -0.003 (-0.53, 0.52) | -0.62 (-1.05, -0.20)** -0.07 (-0.38, 0.24) |  |  |  |
| ***p-value < 0.001, **p-value < 0.01, *p-value < 0.05;  Abbreviations: CRS_s_ = European Position Paper on Rhinosinusitis subjective symptoms definition for CRS classification  ^a^ Ordered probit regression yielded β-coefficients which represented a β-change in z-score of underlying outcome scale; all models adjusted for season, female sex, ASI, migraine status, and binary indicator for whether symptoms taken from questionnaire occurred > 90 days from time of CT scan.  ^b^ Additionally included ASI^2^ and interactions for female sex by ASI and ASI^2^.  ^c^ Additionally included self-reported physician diagnosis of hay fever, age, and interactions for female sex by ASI and by age.  ^d^ Additionally included age, age^2^, Charlson comorbidity index, binary indicator for receipt of Medical Assistance, ASI^2^, and interactions for female sex by ASI and ASI^2^.  ^e^ Additionally included self-reported physician diagnosed hay fever, age, and age^2^.  ^f^ Based on responses to four questions, at baseline, from the ID Migraine questionnaire. | | | | | | |
